# Factors associated with adequate weekly reporting for disease surveillance data among health facilities in Nairobi County, Kenya, 2013

**DOI:** 10.11604/pamj.2016.23.165.8758

**Published:** 2016-04-06

**Authors:** Athman Juma Mwatondo, Zipporah Ng'ang'a, Caroline Maina, Lyndah Makayotto, Moses Mwangi, Ian Njeru, Wences Arvelo

**Affiliations:** 1 Kenya Field Epidemiology and Laboratory Training Program, Ministry of Health, Nairobi, Kenya; 2 College of Health Sciences, Jomo Kenyatta University of Agriculture and Technology, Nairobi, Kenya; 3 Disease Surveillance and Response Unit, Ministry of Health, Nairobi, Kenya; 4 Centre for Public Health Research, Kenya Medical Research Institute, Nairobi, Kenya; 5 US Centers for Disease Control and Prevention, Nairobi, Kenya; 6 Division of Global Health Protection, Center for Global Health, Centers for Disease Control and Prevention, Atlanta, USA

**Keywords:** Surveillance, prevalence, Kenya

## Abstract

**Introduction:**

Kenya adopted the Integrated Disease Surveillance and Response (IDSR) strategy in 1998 to strengthen disease surveillance and epidemic response. However, the goal of weekly surveillance reporting among health facilities has not been achieved. We conducted a cross-sectional study to determine the prevalence of adequate reporting and factors associated with IDSR reporting among health facilities in one Kenyan County.

**Methods:**

Health facilities (public and private) were enrolled using stratified random sampling from 348 facilities prioritized for routine surveillance reporting. Adequately-reporting facilities were defined as those which submitted >10 weekly reports during a twelve-week period and a poor reporting facilities were those which submitted <10 weekly reports. Multivariate logistic regression with backward selection was used to identify risk factors associated with adequate reporting.

**Results:**

From September 2 through November 30, 2013, we enrolled 175 health facilities; 130(74%) were private and 45(26%) were public. Of the 175 health facilities, 77 (44%) facilities classified as adequate reporting and 98 (56%) were reporting poorly. Multivariate analysis identified three factors to be independently associated with weekly adequate reporting: having weekly reporting forms at visit (AOR19, 95% CI: 6-65], having posters showing IDSR functions (AOR8, 95% CI: 2-12) and having a designated surveillance focal person (AOR7, 95% CI: 2-20).

**Conclusion:**

The majority of health facilities in Nairobi County were reporting poorly to IDSR and we recommend that the Ministry of Health provide all health facilities in Nairobi County with weekly reporting tools and offer specific trainings on IDSR which will help designate a focal surveillance person.

## Introduction

According to WHO, the definition of public health surveillance is the continuous, systematic collection, analysis and interpretation of health-related data needed for the planning, implementation, and evaluation of public health practice [1]. Surveillance provides information for effective action [2, 3], and is the backbone of disease control. Poor disease surveillance has been identified as one of the major factors responsible for increasing mortality and morbidity due to communicable diseases [4, 5]. An effective surveillance system has also been associated with reductions in time-to-peak of outbreak [6] and reductions in total cumulative incidence of cases and mortality as well as cases averted [7]. However, late reporting, inaccurate reports or incomplete reports of surveillance data may result in outbreaks going undetected or detected late leading to high morbidity and mortality [8]. The recent outbreak of Ebola Viral Disease in West Africa has demonstrated the importance of an effective national surveillance and response system [9].

The Integrated Disease Surveillance and Response (IDSR) strategy was instituted by the World Health Organization Africa Regional Office in 1998 [10] with an objective to strengthen the availability and use of surveillance data for detecting, reporting, investigating, confirming, and responding to preventable priority diseases as well as other public health events. Implementation of IDSR was a direct response to several large outbreaks with high morbidity and mortality across the African continents, such as cholera and Ebola Viral Disease [11].

In 2005, International Health Regulations (IHR) came into force to prevent, control, and respond to international spread of disease [12]. In the African region, implementation of these regulations is being done through the IDSR platform [13]. Improved surveillance through the IDSR strategy is therefore imperative in assuring IHR compliance.

As of June 2010, 43 countries out of 46 in Africa were at different levels of implementation of the IDSR strategy [14]. Kenya adopted the IDSR strategy in 1998 following the World Health Organization (WHO) Resolution in Harare [10] and implementation began in 2002. Kenya has selected 35 priority diseases for reporting that are epidemic prone, targeted for elimination or eradication, or are of public health importance [15].

In Kenya, IDSR seeks to capture health information of priority communicable and non-communicable diseases for prevention and control by linking communities, health facilities, districts, counties and national levels. Each health facility detects, confirms and records these diseases on specific pre-designed forms using standard case definitions. These reports are then sent to Subcounty Disease Surveillance Coordinator (SDSC) each Monday who aggregates all reports and sends to the national level by Wednesday [15]. Weekly reports from health facilities are transmitted to the Subcounty through various modes including Short Messaging Service (SMS), email, fax or hand delivery of hardcopy report. Each facility must retain a copy of the reporting form and submit the original copy to the Subcounty within the following week. From the Subcounty, the aggregated Subcounty weekly report is sent to the national level via a web based platform. The SDSC, among other duties, is expected to provide feedback on the weekly reporting to health facilities and arrange support supervision in liaison with the Subcounty Health Management Team (SHMT) at least once every three months.

Identifying gaps in the implementation of weekly reporting at health facility level can lead to practical recommendations that can improve integrated disease surveillance among health facilities in Kenya. Although weekly reports submitted to the national level by sub counties (aggregate of sub county facility reports) exceeded the national target of 80%,health facility level weekly reporting for the whole country was low in 2012 at 69% and even lower at 61% in 2013 [16].

In Nairobi County, 63% of health facilities were submitting reports each week in 2013. There has been no assessment of weekly reporting of surveillance data in Nairobi County to determine the quality of reports submitted or to identify factors that might influence adequate reporting.

The objectives of this study were to review the extent of health facilities Nairobi County reporting weekly for IDSR priority diseases, to determine timeliness and completeness of these reports, and to identify enabling factors associated with health facilities meeting the mandated weekly reporting targets.

## Methods

### Study area

Nairobi County, one of 47 counties in Kenya, is Kenya's most populous county with a population of 3.1 million and a population density of 4,509/km2 [17]. Nairobi is Kenya's economic and political capital and is a convergence zone for road, rail and air travel. Jomo Kenyatta International Airport serves about 19,000 passengers daily from Africa, Europe and Asia [18]. Over 60% of the population in Nairobi lives in informal settlements [19] where health and sanitary services are inadequate. Nairobi County has nine administrative sub counties with functional Sub-county Health Management Teams (SHMT) which are under the County Health Management Team (CHMT). Nairobi County has approximately 800 registered health facilities that offer preventive, curative and rehabilitative services [20]. As of June 2013, there were 348 health facilities that were required to meet the mandated weekly reporting targets.

### Study design

We conducted a cross-sectional study of health facilities in Nairobi County between 3rd February and 7th April 2014. Facilities required to report had to be registered by Nairobi County department of Health before June 2013 and offering at least preventive and curative services at an outpatient or inpatient level.

We used stratified random sampling to select health facilities for the study. To form the sampling frame from which we selected health facilities to survey, we listed all 348 health facilities in Nairobi County required to meet the weekly reporting targets for surveillance reporting provided by the nine Subcounty Health Management Teams. The sample size for study of 183 health facilities was determined using the Cochran′s correction formula [21] using the finite population correction (1977). We a precision of 5% and 50% as the assumed proportion of facilities reporting adequately because we published study that demonstrated adequate reporting among health facilities.

Stratification was based on two categories; public health facilities and private health facilities. A random sample from each stratum was drawn in a number proportional to the stratum′s size when compared to the population. These subsets of the strata were then pooled to form the random sample.

A structured questionnaire was used to collect data. The questionnaires were administered in-person at each health facility to the healthcare worker responsible for surveillance or officer-in-charge of reporting activities in each facility. The tool used was partly adopted from the WHO protocol for the assessment of national communicable disease surveillance and response [22] and the Kenyan IDSR Technical guidelines [15]. We assessed facility level factors that enable appropriate reporting which included availability of reporting forms, case definitions, guidelines and hardware such as computers and internet connectivity. We also conducted a retrospective review of hardcopy weekly surveillance reports filled for 12 complete weeks from 2nd September 2013 to 29th November 2013. Available reports for this period were reviewed for completeness of reports and timeliness of report submission.

An adequately reporting health facility was defined as one that submitted 10 or more reports in the twelve-week period. A poorly reporting health facility was defined as one that submitted less than 10 reports in the twelve week period. The cut-off of 10 reports was based on the target of 80% reporting for health facilities in the IDSR technical guidelines. Complete reports were forms with all reportable diseases addressed; including zero reporting for reportable diseases in which no cases were detected. All cells in the reporting forms had to be filled for it to be considered complete. Timely reports were defined as weekly reports submitted by end of each day on Monday of the following week. The date on the reporting form was used to evaluate timeliness. Reports that missed the date were not considered for the analysis of timeliness.

### Data analysis and management

Data were entered, cleaned and analyzed using Epi info version 7(CDC, Atlanta, GA, USA) and stored in Microsoft Access database (Microsoft Office, Seattle, USA). We performed univariate analysis for descriptive statistics followed by bivariate analysis to identify factors that influenced weekly reporting in Nairobi County. We calculated odds ratios and 95% confidence intervals (CI) comparing adequately reporting facilities and poorly reporting facilities. We performed multivariate logistic regression analysis to determine independent factors associated with adequate reporting. P-value < 0.20 was used to select variables for inclusion into the initial multiple logistic regression model. The final model was developed by backwards elimination, dropping the least significant independent variable until all the remaining predictor variables were significant (p-value < 0.05).

### Ethics

We obtained written informed consent from the selected interviewees in each health facility. Prior to starting the study, ethical approval was obtained from Kenyatta National Hospital/University of Nairobi -Ethics and Research Committee (KNH/UON-ERC). Permission was obtained from Nairobi County Health Management Team. We also sought for permission from the health facility leadership before starting the interviews.

## Results

We enrolled 175 health facilities (96% of sampled facilities): 130 (74%) were private health facilities; 45(26%) were public facilities; 126(72%) facilities offered only outpatient services and 49 (28%) facilities offered both outpatient and inpatient care (Table 1). Of the 175 health facilities, 77 (44%) were classified as adequate reporters and 98 (56%) were poor reporters. Seventy one (41%) facilities submitted no reports at all in the twelve week period. Of the eight (4%) facilities that were not enrolled three declined and five didn't have/didn't avail the reporting forms for assessment of reporting. One facility was a public and seven were privately owned.

**Table 1:** Descriptive statistics of 175 health facilities surveyed for weekly IDSR surveillance activities in Nairobi County, Kenya, 2013

Characteristics	n	%
At least one report submitted	104	59.4
**Facility Type**		
Private	130	74.3
Public	45	25.7
**Service Delivery**		
Outpatient only	126	72.0
Outpatient and Inpatient	49	28.0
**Case definitions available**		
Measles	117	66.9
Yellow fever	74	42.3
Acute Flaccid Paralysis	110	62.9
Cholera	80	45.7
Neonatal tetanus	99	56.6
None	40	22.9
Availability of weekly reporting forms (MOH 505)	112	64.0
Stock outs of weekly reporting forms in last 3 months (MOH 505)	71	41.0
Participation in Surveillance review meetings in last 3 months	88	50.3
Designated Surveillance Focal person	106	60.5
Reporting officer Trained in IDSR	97	55.4
IDSR Technical Guidelines Available	11	6.3
Surveillance work plan for 2013 present	11	6.3
Posters with surveillance functions available	70	40.0
Facility supervised in last 3 months	92	52.6
Facility has access to computer devices	118	67.4
Facility has access to Internet	95	54.3
Evidence of data analysis in last 3 months	116	66.3

The preferred mode of reporting by facilities was Short Messaging Service (SMS) 60(58%), followed by hand-delivered hardcopy of the weekly reporting form 40(38%) and by email 4(4%). The median number of reports submitted by adequate reporters was 12(range: 10-12), and 3(range: 0-9) for poor reporters. In evaluating completeness of weekly reports, 87% of the weekly forms were filled to completeness while timeliness of the reports was at 93%. Weekly reporting forms were present in 112 (64%) health facilities at the time of visit while 71(41%) health facilities experienced stock-outs of reporting forms in the three months prior to the visit. Most facilities 118 (67%) had access to at least one computer device (desktops, laptop, tablet or Smartphone) while internet was accessible in 95 (54%) of health facilities. IDSR technical guidelines were found in 11(6.3%) facilities while 116(66.3%) facilities had some evidence of data analysis through displayed trend graphs. Dedicated surveillance persons were found in 106(60.5%) of health facilities, who took full responsibility of surveillance reporting in the facility.

On bivariate analysis, we found facilities that had at least one poster displaying a disease specific case definition had an increased odds being adequate reporters than those without any case definitions (OR 6, 95% CI: 2-17). Increased odds of adequate weekly reporting was also found in facilities with weekly reporting forms present compared to those without weekly reporting forms present (OR28, CI: 9-82), in facilities with health workers trained or sensitized on IDSR compared to facilities without health workers trained on IDSR (OR 11.2, 95% CI: 5-24) and in those facilities that had received quarterly support supervision from Subcounty team compared to those that did not receive quarterly supportive supervision (OR7, 95% CI: 4-14). Having a designated surveillance focal person was also found to have an increased odds of adequate reporting compared to facilities with no designated focal person (OR21, 95% CI: 8-54) as well as in facilities that were conducting data analysis as compared to those that did not conduct data analysis (OR3, 95% CI: 2-6). Facilities that had a posters displaying IDSR functions had a higher odd of adequate reporting as compared to those with no posters displayed in the facility (OR 6.4, 95% CI: 3.3-12.5) (Table 2).

**Table 2:** Bivariate analysis of the determinants of adequate reporting among health facilities in Nairobi County, Kenya, 2013

Characteristics	Adequate Reporters(N=77)		Poor Reporters (N=98)		Odds Ratio	95% C I		p value
	n	%	n	%				
**Facility Type**								
* Public*	19	42%	26	58%	0.9	0.5	1.8	0.917
* Private*	58	45%	72	55%				
**Service Delivery level**								
* Outpatient*	57	45%	69	55%	1.2	0.6	2.34	0.598
* Outpatient and Inpatient*	20	41%	29	59%				
Case definitions available	72	51%	69	49%	6.1	2.2	16.5	<0.001
Availability of weekly reporting forms (MOH 505)	73	65%	39	35%	27.6	9.3	81.7	<0.001
Stock outs of weekly reporting forms in last 3 months (MOH 505)	9	13%	62	87%	0.1	<0.01	0.2	<0.001
Participation in Surveillance review meetings in last 3 months	60	68%	28	32%	5.5	2.9	10.6	<0.001
Designated Surveillance Focal person	71	33%	35	33%	21.3	8.4	54	<0.001
Reporting officer Trained in IDSR	65	67%	32	33%	11.2	5.3	23.6	<0.001
IDSR Technical Guidelines Available	10	91%	1	9%	7.2	1.5	33.8	0.011
Surveillance work plan for 2013 present	8	73%	3	27%	3.7	0.9	14.3	0.062
Posters with surveillance functions available	49	70%	21	30%	6.4	3.3	12.5	<0.001
Facility supervised in last 3 months	60	65%	32	35%	7.3	3.7	14.4	<0.001
Facility has access to computer devices	53	45%	65	55%	1.1	0.6	2.1	0.851
Facility has access to Internet	42	44%	53	56%	1	0.6	1.9	0.927
Evidence of data analysis in last 3 months	61	53%	55	47%	3	1.5	5.9	0.002

Using logistic regression with a backwards elimination strategy, three significant predictors were found to be independently associated with adequate reporting. These factors included a facility having the weekly reporting forms as compared to those that did not have the forms (Adjusted OR [AOR] 19, 95% CI: 6-65), a facility having a designated surveillance focal person as compared to those without a designated focal person (AOR7, 95% CI: 2-20) and availability of posters with IDSR functions in the facility as compared to facilities that did not have these IDSR posters (AOR8, 95% CI: 2-12) (Table 3).

**Table 3:** Summary of factors independently associated with being an adequate reporter among health facilities on multivariate analysis, Nairobi County, Kenya, 2013

Characteristics	Crude Odds Ratio	Adjusted Odds Ratio	95% C I		p value
Availability of weekly reporting forms (MOH 505)	27.60	19.00	5.56	64.86	<0.001
Designated Surveillance Focal person	21.30	7.09	2.48	20.28	<0.001
Posters with surveillance functions available	6.40	4.84	1.90	12.30	0.001

## Discussion

This cross sectional survey looking at surveillance performance showed that adequate weekly reporting to IDSR system in Nairobi County was low and below the target of 80% given in the WHO IDSR guidelines. To further explain these findings, we found three factors that were independently associated with promoting adequate reporting in Nairobi County: a facility having weekly reporting forms, a facility with a dedicated surveillance focal person and a facility with posters of IDSR functions displayed.

One characteristic found to promote adequate reporting for surveillance was a facility having the weekly reporting forms also entitled as MOH 505 forms. These forms are used to enter weekly data prior to submission to the Subcounty level. Facilities that reported frequent stock-outs three months prior to the visit were also found to report poorly. The proportion of available forms in health facilities in Nairobi County was higher than the 76% found in another cross-sectional study in Nigeria [23]. In another facility study in Nigeria, lack of adequate surveillance forms was also considered a significant factor related to poor surveillance reporting [24]. Because the standard surveillance data recording and reporting at the health facility level is currently paper-based, the provision of these weekly reporting forms is paramount in ensuring surveillance data recording and reporting is consistent.

Another important factor associated with adequate reporting was a facility having a designated surveillance focal person. Very few published studies have demonstrated this finding in the African region. In other descriptive studies, this phenomenon has been related to staff having too high a work load to find time to report surveillance data [25], staff having tasks that compete with reporting surveillance activities, or under staffing at the health facility [23, 26]. In such situations, one health worker may manage a whole health facility and they may prioritize patient care while surveillance reporting suffers [27]. Having a designated surveillance focal person makes surveillance reporting the core responsibility of the officers’ work in the facility. Predictably, this is likely to increase the level of surveillance reporting. Surveillance focal persons were present in 72% of all health facilities studied, which was slightly higher than 60% found in a 2009 IHR core capacity assessment conducted in Kenya [28]. Having a surveillance focal person and the focal person trained in IDSR were closely related, with 95% of the designated surveillance focal persons trained in IDSR. This is an expected finding because training helps designate a focal person and likely explains why training in surveillance was not independently associated with adequate reporting.

Having posters illustrating IDSR guidelines/functions was also associated with improved reporting. Although having standard case definitions present at the facility was not independently associated with adequate reporting, posters displaying the surveillance functions that include case definitions may also serve as reminders which improve surveillance reporting. About two thirds of the facilities visited had a form of data analysis on surveillance with displayed and updated trend graphs. This was substantially higher than earlier studies in Tanzania at 32% [29] and surveillance evaluation study in Nigeria at 19% [30]. Analysis and interpretation of data at the health facility level allows for early detection and prompt response to outbreaks.

Additionally, we also found that reporting of weekly surveillance data from health facilities to the Sub County was mostly through SMS followed by hand delivery, and mail reporting was least popular. The SMS usage for reporting was much lower than 92% SMS reporting found in a 2013 nationwide data quality audit among health facilities [31]. This could be because of the close proximity of health facility in Nairobi County to the Sub County offices and ease of transportation allowed for hand delivery of surveillance reports. Reporting timeliness and completeness were satisfactory and surpassed the mandated target of 80% for those facilities that were reporting. Timeliness of reporting was 93%, which is higher than 65% reported in an assessment of weekly reporting in Uganda [25] and 47% reported in surveillance evaluation in Tanzania [29]. Completeness of reports (87%) also met standards recommended by WHO and reduces the likelihood of underreporting. Though SMS reporting is faster and less time consuming resulting in high usage among peripheral health facilities, this form of reporting is not standardised and could diminish completeness and quality of reports submitted.

Lastly, only about half of heath facilities were supervised in three months by the Subcounty team. Supervised facilities were seven times more likely to be adequate reporters. The proportion of facilities supervised was higher than 18% found in Iraq [32] but lower than 70% found in Eritrea [33]. Clearly, improved support supervision of health facilities could result in enhanced reporting of priority diseases and surveillance performance in general.

Our study findings were subject to a few limitations. Timeliness of reports was calculated using the date stated on the reporting form. It is possible that the date may not entirely reflect the true day a report was submitted. A report could be submitted late but the date stated could be timely. In addition, the study was conducted in one county and therefore cannot be generalised to the rest of the country. Our study was conducted in an urban setting and we feel further studies conducted in rural or mixed settings in different counties could illuminate more information on the challenges of weekly reporting among health facilities in Kenya.

## Conclusion

There was suboptimal IDSR priority disease reporting among majority of health facilities in this urban county, which poses a risk on timely outbreak detection and responses. Fortunately, facilities that did report were doing so in a timely fashion and submitted complete reports. We found that the availability of reporting forms, having a dedicated surveillance focal person and availability of poster displaying IDSR functions critical in promoting adequate reporting. No difference in reporting was noted between public and private health facilities. Therefore, in similar urban settings, we recommend that all health facilities are provided with weekly reporting tools, IDSR technical guidelines and specific trainings in IDSR, and designate a dedicated surveillance focal person, if not done so already. Furthermore, IDSR function posters/guidelines should be made readily available to improve weekly reporting.

### What is known about this topic

- Disease surveillance is the backbone of disease control and Kenya has made good progress in IDSR implementation with focal persons in most districts and electronic reporting at district level;
- Health facilities are the primary sources of disease data but their reporting rates have been below the target of 80% reporting.

### What this study adds

- This study confirms that health facilities are reporting poorly to IDSR system in Nairobi County, posing a serious threat to early disease detection. Yet, the facilities that report, do so consistently, in a timely and accurate manner;
- It demonstrates the potential reasons for the poor reporting as unavailability of reporting forms, lack of a dedicated surveillance focal person and inadequate support supervision.
